# Brain Functional Connectivity Through Phase Coupling of Neuronal Oscillations: A Perspective From Magnetoencephalography

**DOI:** 10.3389/fnins.2019.00964

**Published:** 2019-09-12

**Authors:** Laura Marzetti, Alessio Basti, Federico Chella, Antea D'Andrea, Jaakko Syrjälä, Vittorio Pizzella

**Affiliations:** ^1^Imaging and Clinical Sciences, Department of Neuroscience, University of Chieti-Pescara, Chieti, Italy; ^2^Institute for Advanced Biomedical Technologies, University of Chieti-Pescara, Chieti, Italy

**Keywords:** magnetoencephalography, functional connectivity, brain networks, phase coupling, brain rhythms

## Abstract

Magnetoencephalography has gained an increasing importance in systems neuroscience thanks to the possibility it offers of unraveling brain networks at time-scales relevant to behavior, i.e., frequencies in the 1–100 Hz range, with sufficient spatial resolution. In the first part of this review, we describe, in a unified mathematical framework, a large set of metrics used to estimate MEG functional connectivity at the same or at different frequencies. The different metrics are presented according to their characteristics: same-frequency or cross-frequency, univariate or multivariate, directed or undirected. We focus on phase coupling metrics given that phase coupling of neuronal oscillations is a putative mechanism for inter-areal communication, and that MEG is an ideal tool to non-invasively detect such coupling. In the second part of this review, we present examples of the use of specific phase methods on real MEG data in the context of resting state, visuospatial attention and working memory. Overall, the results of the studies provide evidence for frequency specific and/or cross-frequency brain circuits which partially overlap with brain networks as identified by hemodynamic-based imaging techniques, such as functional Magnetic Resonance (fMRI). Additionally, the relation of these functional brain circuits to anatomy and to behavior highlights the usefulness of MEG phase coupling in systems neuroscience studies. In conclusion, we believe that the field of MEG functional connectivity has made substantial steps forward in the recent years and is now ready for bringing the study of brain networks to a more mechanistic understanding.

## Introduction

In the last decades, systems neuroscience has made it clear that brain functioning requires the cooperation of several spatially separated brain regions to allow for integrative functions (e.g., vision, audition), as well as for higher order functions (e.g., understanding of actions), for a review see e.g., Rizzolatti et al. (2018). The reliable estimation of this cooperation, i.e., of functional connectivity between brain areas, is thus of primary importance to disclose the physiological and pathological organization of the human brain. To this end, several non-invasive imaging techniques and novel analysis methods have contributed to the examination of whole-brain functional connectivity patterns: functional Magnetic Resonance (fMRI) and functional Near Infrared Spectroscopy (fNIRS) have investigated the level of co-activation between brain regions as a proxy for functional communication (e.g., Smith et al., 2013; Wang et al., 2017), while electrophysiological techniques such as ElectroEncephaloGraphy (EEG) and MagnetoEncephaloGraphy (MEG), have characterized both the level of co-activation between brain regions and the coupling of their respective signals, i.e., the statistical relationship between time-series of neuronal signals. A recent review on the theory and algorithms of electrophysiological brain connectivity analysis can be found in He et al. (2019).

In this review we will focus on MEG, i.e., the measurement of the magnetic field generated by neural currents. Indeed, MEG has gained an increasing importance in systems neuroscience as testified by the impact of MEG related publications (Baillet, 2017). Magnetoencephalography, being able to track neuronal activity without the filtering effect of the neuro-vascular coupling (Singh, 2012) can provide direct information about neuronal activity and functional connectivity. In the last decade, MEG functional connectivity has contributed to reinforce the concept of Resting State Networks (RSNs) as defined by fMRI (Deco and Corbetta, 2011) by assessing the correlation between the Blood-Oxygen-Level-Dependent (BOLD) time-series of two brain regions. In MEG, initially Amplitude Envelope Correlation (AEC) has been used to assess the level of co-activation between MEG signals of different brain regions. Specifically, MEG AEC has been calculated as the correlation of the slow temporal fluctuations (envelope) of the orthogonalized MEG signals (for a review see O'Neill et al., 2015). Moreover, MEG is able to track neuronal activity at its characteristic time scale, i.e., milliseconds, and it is thus ideally suited to assess the faster dynamics of the different brain areas as well as of their functional connectivity. Indeed, this characteristic makes MEG also able to investigate, with high spectral resolution, neural oscillations which are known to subserve brain connectivity and to play important roles in cognitive processes (Varela et al., 2001; Engel et al., 2013). In more detail, it has been hypothesized that only coherently oscillating neuronal groups can interact effectively. This hypothesis, namely Communication Through Coherence (CTC; Fries, 2005), is grounded in the fact that neural oscillations are associated to neuronal excitability fluctuations: two neuronal groups can communicate only when they share the same excitability state, with a possible time lag between the two that takes into account the speed of traveling signals (Bastos et al., 2015; Fries, 2015). Thus, long range connectivity can be assessed through the characterization of phase-phase coupling (hereinafter simply referred to as phase coupling) between MEG signals which has also been related to the concept of RSNs (e.g., Marzetti et al., 2013).

This review aims at introducing the different methods used to assess connectivity in MEG, with emphasis on methods based on phase coupling. We will discuss the advantages of studying brain connectivity starting from neural sources, as estimated from MEG, over studying brain connectivity directly from the measured signals. MEG has been often considered similar to EEG since both are related to the electromagnetic field generated by the currents flowing within neurons and in the surrounding medium, i.e., the brain volume. Indeed, while sharing the same elementary neuronal phenomena, each of the two techniques has its own strengths as summarized in Lopes da Silva (2013), and the physics laws that describe how these currents translate into magnetic field and electric potential show the differences between the two techniques (Hämäläinen et al., 1993). Among others, the lower sensitiveness of MEG to the properties of the conducting medium (Vorwerk et al., 2014; Stenroos and Nummenmaa, 2016) and the need for a reference signal in EEG (Chella et al., 2016a, 2017; Van de Steen et al., 2019) imply an important advantage of MEG over EEG in the assessment of brain functional connectivity.

This review is organized as follows. Firstly, the principles of MEG, including the methods used to estimate brain activity and its relevance to neuronal oscillations, will be revised. Secondly, an extensive review of the methods commonly used to assess functional connectivity will be presented, in which the different methods are classified as undirected or directed according to their outputs, univariate or multivariate, and same-frequency or cross-frequency according to their inputs. Finally, the use of phase coupling methods on MEG data in the context of resting state, visuospatial attention and working memory will be presented. Table 1 clarifies the terminology used throughout this review.

**Table 1:** List of terms and definitions used.

**Term**	**Definition**
Magnetoencephalography (MEG)	MEG is a non-invasive neuroimaging technique that can be used to detect extremely weak magnetic fields generated by spatially aligned neurons that are simultaneously activated. The measured MEG signal originates from postsynaptic currents in the apical dendrites of pyramidal neurons.
Neural oscillations	Assemblies of neurons influence each other through excitatory and inhibitory synaptic connections which leads to rhythmic activation and inhibition of neurons in the network. The rhythmic activity is reflected in oscillating signals that can be measured outside of the scalp with EEG and MEG.
Phase	The phase of periodic oscillation, i.e., an oscillation which repeats itself exactly after one period (e.g., sinusoidal wave) indicates the fractional portion of the period that has been completed. The phase is typically expressed as an angle spanning a whole turn (2π) as the oscillation goes through a period. In the context of neural oscillations, the phase reflects the excitability state of the neurons and therefore the phase influences the discharge times of the neurons in the network.
Functional connectivity	Functional connectivity refers to statistical associations or temporal correlations between two or more anatomically distinct brain regions.
Phase coupling	The term phase coupling refers to the relationship between oscillation phases in different brain regions. More specifically, in this work, the term phase coupling is used to indicate the presence of peaks in the distribution of the phase difference across time or signal realizations (e.g., trials, or different segments into which continuous signals can be divided). Phase coupling is considered to be a fundamental neural mechanism that supports neural communication, neural plasticity and it is considered relevant for many cognitive processes. In the literature also the term *phase synchronization* is used as a synonym of phase coupling.
Communication through Coherence (CTC)	CTC refers to the concept that only coherently oscillating neuronal groups can interact efficiently. The concept is grounded in the fact that neural oscillations are associated to neuronal excitability fluctuations. Only when inputs from presynaptic group consistently arrive to the postsynaptic group at the time of high input gain (at appropriate phase) the two groups can effectively communicate. This requires coherence between the pre- and postsynaptic groups since otherwise the inputs arrive at random phases of excitability state and will have less impact on the postsynaptic neurons.

## Magnetoencephalography

The aim of this paragraph is to briefly review the major characteristics of the MEG signal, including its origin, the identification of brain sources from the measured MEG signal, and the potential of MEG to detect neuronal oscillations and to investigate functional connectivity.

Beyond these aspects, we want to emphasize here that the unique features of MEG have made this technique more and more attractive to researchers in order to answer neuroscientific as well as clinical questions (Baillet, 2017). In parallel, the need for guidelines for the acquisition and analysis of MEG data has emerged. To instruct newcomers, as well as to standardize procedures across MEG laboratories, good practice papers have been recently published for both basic (Gross et al., 2013) and clinical neuroscience (Burgess et al., 2011; Hari et al., 2018). To facilitate the analysis, several open-source software packages are available to the user, the most widely used being: Brainstorm (Tadel et al., 2011), FieldTrip (Oostenveld et al., 2011), MNE (Gramfort et al., 2014), and SPM8 (Litvak et al., 2011). Additionally, a multimodal extension of the Brain Imaging Data Structure (BIDS), originally proposed to standardize MRI data formats (MRI-BIDS), has been proposed for MEG data (MEG-BIDS; Niso et al., 2018). This will allow a smoother data harmonization as well as to reduce sharing efforts across the MEG community.

### Origin of MEG Signal

Nowadays, it is commonly agreed that MEG signals originate mostly from postsynaptic currents in the apical dendrites of pyramidal neurons in the cortical mantle. Maxwell's equations in their quasi-static approximation describe how these currents translate into magnetic field (Hämäläinen et al., 1993). The MEG challenge lies into measuring such an extremely weak magnetic field, about one million times smaller than the Earth's magnetic field, in an often magnetically noisy environment due to nearby large metal objects or strong electric currents. Typically, the simultaneous activation of about tens of thousands of spatially aligned nearby neurons (i.e., a neuronal pool) is needed to generate a detectable MEG signal.

All of the currently operating MEG systems are based on superconducting magnetometers, namely SQUIDs (superconducting quantum interference device). These sensors feature an exquisite sensitivity to pick up the weak neuromagnetic fields the bandwidth of which spans from few kHz down to almost DC (Baillet, 2017). Moreover, all MEG systems operate in a magnetic shielded room to reduce environmental magnetic noise. Recently, the development of new sensors operating at room temperature, namely Optically Pumped Magnetometers (OPMs), have open new opportunities for the widening of MEG applications. These devices, although still suffering from higher noise and limited bandwidth with respect to SQUIDs, offer the possibility to record MEG using a wearable device in a more ecological setting (Boto et al., 2018; Iivanainen et al., 2019).

### Source Space MEG

MEG sensor signals arise from the mixture of the activity of several neuronal pools. In fact, each MEG sensor measures, with different weights, the signal generated by all neuronal pools active at a given time. This results in the overlap, at sensor level, of the fields generated by each pool. Even if one would unrealistically assume that only one pool is active, and thus no actual brain connectivity is present, a fake connectivity profile between the sensors would be detected as the result of all sensors measuring the activity of the one brain source. Thus, assessing true brain connectivity directly from MEG signals at sensor level is challenging (Schoffelen and Gross, 2009).

A prerequisite to reliably assess functional connectivity is to rely on source space data, i.e., the activity of each neuronal pool. In fact, the estimation of functional connectivity from sensor space data, i.e., from the activities measured by the sensors outside the head, is not only prone to the above described fake connectivity profile but is also very difficult to be interpreted in terms of the underlying functional connectivity between brain regions given that the same connectivity profile can be generated by different configurations of interacting sources. Unfortunately, the estimation of the activities of brain regions requires the solution of an ill-posed electromagnetic inverse problem (Ilmoniemi and Sarvas, 2019). In practice, there exist reasonable approximations incorporating prior knowledge (e.g., from fMRI) and constraints (e.g., minimum norm) that make this issue less severe and allow the use of MEG in brain imaging and in the study of brain networks.

The solution to the MEG inverse problem requires a forward model, which gives the mathematical relationship between the brain currents (sources) and the measured magnetic field. The forward model includes models for the sources, the geometry of the conducting medium (i.e., the head), and the MEG sensors. Besides the intrinsic non-uniqueness of the electromagnetic inverse problem solution and the correctness of *a priori* assumptions and constraints used to make the solution unique, the attainable accuracy in the solution highly depends on the accuracy of these models. The influence of the head model has been investigated in a number of studies (e.g., Güllmar et al., 2010; Stenroos et al., 2014; Vorwerk et al., 2014). The distinction between white and gray matter and the inclusion of the cerebrospinal fluid have been shown to considerably improve forward model accuracy (Vorwerk et al., 2014; Stenroos and Nummenmaa, 2016). To date, anatomically realistic and sufficiently detailed head models can be constructed from the segmentation of structural head images (usually MRI or computed tomography scans; for guidelines, see Vorwerk et al., 2014). Nonetheless, the forward model accuracy is still limited by approximations in geometry structures, uncertainty in conductivity values (Akhtari et al., 2002; Dannhauer et al., 2011), as well as errors in head-to-sensor co-registration (Adjamian et al., 2004; Troebinger et al., 2014; Chella et al., 2019).

The estimates of neural activity are usually obtained by either a localization approach or an imaging approach. The former assumes that brain activity at a given time point is generated by a limited number of neuronal pools, each represented by an equivalent current dipole (ECD), the location, orientation, and strength of which need to be estimated. Numerical approaches based on least-squares techniques are usually used to this purpose. The latter, i.e., the imaging approach, aims at estimating the overall distribution of neural activity by discretizing the brain, and thus is more suitable for whole brain functional connectivity estimation. Typically, a grid of elementary sources (dipoles), fixed in location and, possibly, in orientation, in the brain volume or limited to the cortical gray matter surface is used: the inverse problem solution for imaging approaches results in the estimation of the magnitude of all these elementary sources. Among imaging approaches, several strategies have been developed, including distributed source imaging methods (e.g., least-squares techniques such as minimum norm estimation approaches), scanning methods, and spatial filter methods. For reviews on the different imaging methods see Baillet et al. (2001); He et al. (2018); Ilmoniemi and Sarvas (2019).

The different source imaging methods are all characterized by a source-leakage effect, namely that the measured activity coming from a single elementary source is projected onto several nearby sources depending on the point spread function of the specific imaging method (Hauk et al., 2011). Source-leakage may induce a fake connectivity profile, usually referred to as “artificial connectivity” (Palva and Palva, 2012). This effect can be mitigated by using functional connectivity methods which exclude zero-phase correlations between source signals (see e.g., Marzetti et al., 2008; Nolte et al., 2009; Brookes et al., 2011a). Similarly, a priori selecting regions-of-interest as seeds for connectivity analysis, as opposed to performing whole-brain estimation, can provide misleading results in terms of “spurious connectivity” (Palva and Palva, 2012) as the activity of the sources not selected as seeds may leak into the estimated activity of the selected seeds (Hari and Parkkonen, 2015).

**Figure 1:**
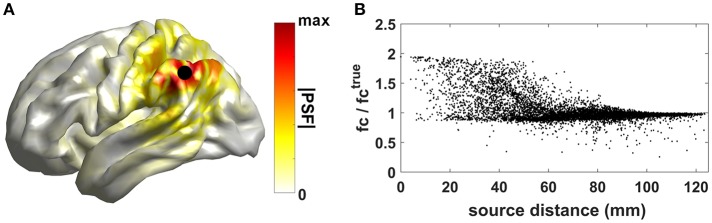
Effects of source leakage on connectivity estimates. **(A)** Cortical map for the absolute value of the Point Spread Function (PSF) of source 1. **(B)** Ratio between the estimated functional connectivity values and the true value (i.e., the one obtained directly from the generated source time sources) as a function of the distance between sources 1 and 2.

To show an example of this effect, 2-min of synthetic MEG recordings, sampled at 512 Hz, were simulated by using the head model and MEG sensor layout of one subject taken from the Human Connectome Project dataset (Larson-Prior et al., 2013). The time series for two interacting dipolar sources in the cortex, with orientation perpendicular to the local cortical surface, were generated as follows: the time series of source 1 was sampled from a Gaussian distribution; the time series of source 2 was set to a Finite-Impulse-Response (FIR) filtered version of the time series of the first source; the FIR filter coefficients were randomly drawn from a standard normal distribution and a filter order *P* = 5 was used. The location of source 1 was kept fixed (black dot in panel A of Figure 1), while the location of source 2 was varied across all the remaining cortical locations. The source time series were then projected to the sensor level by solving the MEG forward problem (Nolte, 2003). Uncorrelated sensor noise was added to sensor signals. From the synthetic recordings, source time series were reconstructed by using the eLORETA inverse approach (Pascual-Marqui et al., 2011). In Figure 1A, we show the cortical map for the absolute value of the eLORETA Point Spread Function (PSF) of source 1, a measure of the degree to which the activity originating from a single elementary source leaks into nearby sources.

Functional connectivity between reconstructed source time series was then estimated by using coherence (see section Methods to assess brain connectivity based on phase coupling for the mathematical description of the method). Artificially inflated coherence estimates can be observed in Figure 1B where the ratio between the estimated functional connectivity values and the true value (i.e., the one obtained directly from the generated source time sources) as a function of the distance between sources 1 and 2 is shown. Values larger than 1 indicate inflated functional connectivity values due to zero-lag correlations induced by source leakage. Such an effect is more relevant when the distance between the two interacting sources is small.

### MEG, Neuronal Oscillations, and Functional Connectivity

**Figure 2:**
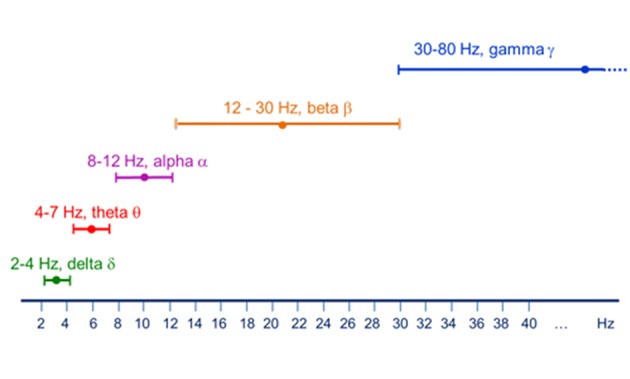
Major brain rhythms as classified by their frequency span.

The activity of neuronal pools features several oscillatory bands with frequencies ranging from about 0.05 Hz up to 500 Hz (Buzsáki and Draguhn, 2004). These frequencies are classified into several frequency bands. Figure 2 below shows one typical classification scheme, although finer grained classifications are also possible (e.g., low alpha/high alpha as well as low gamma/high gamma).

The different frequencies are related to the different properties of the physical architecture of neuronal networks as well as to the speed of neuronal communication limited by axon conduction and synaptic delays. Indeed, higher frequency bands are characteristic of smaller spatial scales, while lower frequencies are distinctive of large-scale networks (von Stein et al., 2000; Florin and Baillet, 2015). Magnetoencephalography, thanks to its exquisite temporal resolution, excels in picking up this rhythmic brain activity and is thus an ideal tool to study local oscillatory activity.

Modulations of local oscillatory activity can be observed as a consequence of task execution or stimulus presentation in a variety of conditions. One of the most prominent examples is provided by motor execution in which the oscillatory beta power in the primary motor cortex is reduced during the movement in comparison to the preceding period (ERD, Event Related Desynchronization) while it is enhanced after the movement (ERS, Event Related Synchronization, i.e., beta rebound), see Pfurtscheller and Lopes da Silva (1999) for details, or section “Magnetoencephalography as a tool for imaging brain activity” in Pizzella et al. (2014) and references therein. A modulation of local oscillatory activity shared by two brain regions is often considered as a marker of functional connectivity. In fact, Amplitude Envelope Correlation approaches have been extensively used to disclose brain functional connectivity during task (Brookes et al., 2011a) as well as at rest (de Pasquale et al., 2010; Brookes et al., 2011b). However, a more physiologically oriented probe on brain functional connectivity can be identified by phase-based metrics under the CTC hypothesis (Fries, 2005), that states that only coherently oscillating neuronal groups can interact effectively. Thus, MEG can be an ideal tool to assess functional connectivity by investigating the phase relationship between two (possibly) interacting areas. This approach will be discussed extensively in the following section.

## Methods to Assess Brain Connectivity Based on Phase Coupling

This section provides an overview of the most widely used methods for assessing brain connectivity based on phase coupling between neural oscillations. The notion of phase is usually associated to a periodic oscillation, i.e., an oscillation which repeats itself exactly after one period (e.g., a sinusoidal wave). For a periodic oscillation, the phase indicates the fractional portion of the period that has been completed. It is typically expressed as an angle spanning a whole turn (2π) as the oscillation goes through a period. Of note, the value of the phase is meaningless if the origin of the oscillation has no physical meaning (e.g., a trigger or a stimulus). Nonetheless, the phase difference between two oscillations is always well-defined, since the dependence on the origin is implicitly canceled out in the computation of the difference. Indeed, the phase difference can be used to assess phase couplings between oscillations, as discussed below.

**Figure 3:**
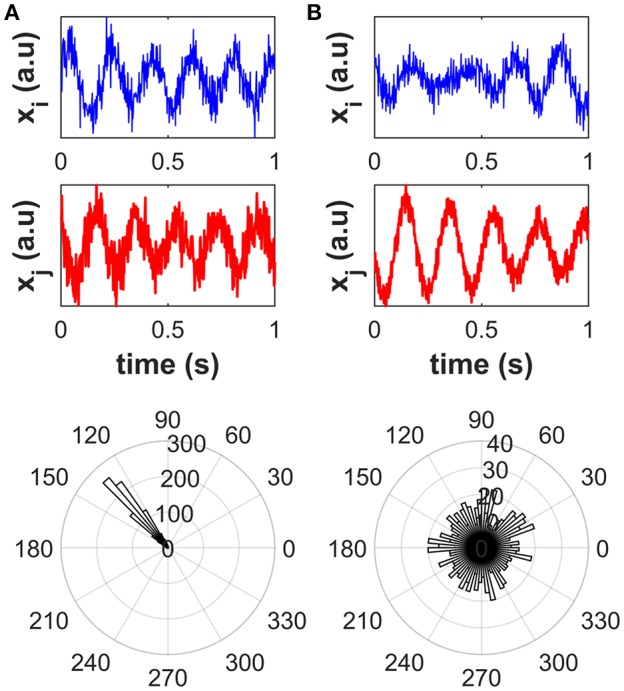
Distribution of the circular phase difference across signal realizations for phase-coupled signals **(A)** and independent signals **(B)**. Top plots: an illustrative example of signal realization. Bottom plots: polar histogram for the distribution of the circular phase difference. A narrow peak in the circular phase difference distribution can be observed in the case of phase-coupled signals, as opposed to the case of independent signals.

The concept of phase coupling has been widely discussed in the literature (e.g., Rosenblum et al., 1996; Pascual-Marqui, 2007a; Stam et al., 2007). In this work, the term “phase coupling” between two signals is meant as the presence of peaks in the distribution of the phase difference across time or signal realizations (e.g., trials, or different segments into which continuous signals can be divided), that reflect preferred values of the phase difference irrespective of the signal amplitudes (see Figure 3). To clarify this concept, we simulated two scenarios depicted in Figure 3. For scenario 1 (Figure 3A), we simulated one-thousand realizations, each of 1-s length and sampled at 512 Hz, of two phase-coupled signals *i* and *j* as described in the next sentences. The time series of a 5-Hz oscillator was generated by band-pass filtering white Gaussian noise around 5 Hz with 1 Hz bandwidth. The time series of the signal *i*, i.e., *x*_*i*_(*t*), was set to the time series of the 5 Hz oscillator while the time series of the signal *j* was set to a time-delayed copy of the time series of signal *i*, i.e., *x*_*j*_(*t*) = *x*_*j*_(*t* − τ), with a time delay τ = 75 ms. Uncorrelated white Gaussian noise was finally added to both the generated signals. For each signal realization, the circular phase difference between signals *i* and *j* was calculated as the phase of their complex-valued coherency (see section Univariate methods for mathematical details). For scenario 2 (Figure 3B) the two signals *i* and *j* were independently generated as two 5-Hz oscillators. A narrow peak in the circular phase difference distribution can be observed in the case of phase-coupled signals (A), as opposed to the case of independent signals (B).

In the following paragraphs, the different connectivity methods will be classified as frequency-specific or cross-frequency phase-coupling methods, depending on whether they allow investigating the phase coupling at the same or different frequencies, respectively. Moreover, they will be divided into univariate and multivariate methods; the formers are designed to assess connectivity from two univariate (or scalar) time series, while the latter take as input multivariate (or vector) time series (see also Table 2).

**Table 2:** List of phase coupling measures.

**Connectivity measure**	**Abbreviations**	**Phase coupling type**	**Class**	**Directionality**	**Artifactual detections due to zero-lag coupling**	**References**	**Software**
Coherency	C	Same-frequency	Univariate	Undirected	Yes	Brillinger, 1981	Brainstorm; FieldTrip; MNE; METH
Phase locking value	PLV	Same-frequency	Univariate	Undirected	Yes	Lachaux et al., 1999	Brainstorm; FieldTrip; MNE; METH
Imaginary part of coherency	ImCoh	Same-frequency	Univariate	Undirected	No	Nolte et al., 2004	Brainstorm; FieldTrip; MNE; METH
Lagged coherence	ρ^2^	Same-frequency	Univariate	Undirected	No	Pascual-Marqui, 2007a	Brainstorm; LORETA-KEY; METH
Imaginary part of phase locking value	iPLV	Same-frequency	Univariate	Undirected	No	Palva and Palva, 2012	
Phase lag index	PLI	Same-frequency	Univariate	Undirected	No	Stam et al., 2007	MNE; METH
Weighted phase lag index	wPLI	Same-frequency	Univariate	Undirected	No	Vinck et al., 2011	FieldTrip; MNE
Phase slope index	PSI	Same-frequency	Univariate	Directed	No	Nolte et al., 2008	FieldTrip; METH
Phase transfer entropy	pTE	Same-frequency	Univariate	Directed	No	Lobier et al., 2014	Brainstorm
Multivariate interaction measure	MIM	Same-frequency	Multivariate	Undirected	No	Ewald et al., 2012	METH
Multivariate lagged coherence	*P*	Same-frequency	Multivariate	Undirected	No	Pascual-Marqui, 2007b	LORETA-KEY
Multivariate phase slope index	MPSI	Same-frequency	Multivariate	Directed	No	Basti et al., 2018	
n:m phase locking value	PLV*^*n*:*m*^*	Cross-frequency	Univariate	Undirected	Yes	Palva et al., 2005	
Bicoherency	Bic	Cross-frequency	Univariate	Undirected	Yes	Nikias and Petropulu, 1993	
Antisymmetric part of bicoherency	aBic	Cross-frequency	Univariate	Undirected	No	Chella et al., 2016b	

*For each connectivity methods we indicate: (i) the abbreviation used in this paper; (ii) whether it investigates same- or cross-frequency phase coupling; (iii) whether it is either a univariate or a multivariate connectivity measure; (iv) whether it is directed or undirected connectivity methods; (v) whether or not the method handles the issue induced by the artificial zero-lag correlation; (vi) a reference publication for the method; (vii) main software toolbox in which the measure is implemented: Brainstorm Tadel et al., 2011; FieldTrip Oostenveld et al., 2011; LORETA-KEY (http://www.uzh.ch/keyinst/loreta.htm#_Toc391372607); METH (https://www.uke.de/english/departments-institutes/institutes/neurophysiology-and-pathophysiology/research/working-groups/); MNE Gramfort et al., 2014*.

### Frequency-Specific Phase Coupling Methods

#### Univariate Methods

Let us denote by *x*_*i*_(*t*) and *x*_*j*_(*t*) the time series of the signals *i* and *j*, which can be, e.g., the estimated activities of two distinct elementary sources. The cross-spectrum between two signals *i* and *j* at a given frequency *f* is defined as

$$\begin{array}{l} S_{ij}\left(f\right):=\langle X_{i}\left(f\right)\;X_{j}^{*}(f)\rangle , \end{array}$$

where *X*_*i*_(*f*) and *X*_*j*_(*f*) are the Fourier transforms of *x*_*i*_(*t*) and *x*_*j*_(*t*), respectively, (∙)^*^ denotes the complex conjugate, and 〈∙〉 denotes the expectation value. The latter is usually evaluated as the average across a sufficiently large number of signal realizations (or segments) under ergodicity assumption.

The *coherency* is a normalized version of the cross-spectrum (Brillinger, 1981; Rosenberg et al., 1989; Halliday et al., 1995), i.e.,

$$\begin{array}{l} C_{ij}\left(f\right):=\frac{S_{ij}\left(f\right)}{\sqrt{S_{ii}\left(f\right)\;S_{jj}\left(f\right)}}, \end{array}$$

the absolute value of which, termed coherence, is a measure of the phase coupling between signals at a given frequency. This can be seen by representing the complex-valued Fourier transforms in terms of their amplitudes *A*_*i*_(*f*) and phases θ_*i*_(*f*), i.e., $X_{i}\left(f\right)={\;A}_{i}\left(f\right)\;e^{\iota \theta_{i}\left(f\right)}$ and $X_{j}\left(f\right)={\;A}_{j}\left(f\right)\;e^{\iota \theta_{j}\left(f\right)}$, where ι denotes the imaginary unit. Using this notation, coherence has the following form:

$$\begin{array}{l} |C_{ij}\left(f\right)|:=\left|\frac{\langle A_{i}\left(f\right){\;A}_{j}\left(f\right)\;e^{\iota \;{\Delta \theta }_{ij}\left(f\right)}\rangle }{\sqrt{\langle A_{i}^{2}\left(f\right)\rangle \;\langle A_{j}^{2}\left(f\right)\rangle }}\right|, \end{array}$$

where the argument of the exponential function contains the phase difference between signals *i* and *j*, i.e., Δθ_*ij*_(*f*) = θ_*i*_(*f*) − θ_*j*_(*f*) ∈ [−π, π]. It turns out that coherence is the absolute value of the weighted average of $e^{\iota \;{\Delta \theta }_{ij}\left(f\right)}$ across signal realizations, where the weights are a function of the amplitudes. If the signals are independent, the phase difference varies randomly across realizations and the coherence vanishes. If the signals are phase-coupled, the phase difference fluctuates around some constant value, and the coherence is non-vanishing.

If the amplitude weights in the above equation are omitted, we get the mean resultant length of the phase difference (i.e., the absolute value of the average of $e^{\iota \;{\Delta \theta }_{ij}\left(f\right)}$ across signal realizations), which is called *phase-locking-value* (PLV; Lachaux et al., 1999), i.e.,

$$\begin{array}{l} {\text{PLV}}_{ij}\left(f\right):=\left|\langle e^{\iota \;{\Delta \theta }_{ij}\left(f\right)}\rangle \right|. \end{array}$$

Alternative formulations for the PLV rely on the instantaneous phase difference between signals, which can be obtained from, e.g., a wavelet or Hilbert transform (Tass et al., 1998, Lachaux et al., 1999; Colclough et al., 2016).

A limitation on the use of the above measures for the assessment of phase coupling between reconstructed source signals is that they are affected by artificial zero-lag correlations, such as the ones induced by source leakage, which inflate the estimated values. In the last two decades, a number of measures have been proposed to mitigate this issue. Nolte et al. (2004) suggested the use of the *imaginary part of coherency* (ImCoh)

$$\begin{array}{l} {\text{ImCoh}}_{i}j\left(f\right):=ℑ\left(C_{i}j\left(f\right)\right), \end{array}$$

since it requires a phase difference between signals to be non-vanishing, and thus it does not lead to artificial phase coupling detections due to zero-lag correlations due to e.g. source leakage. An alternative approach was proposed by Pascual-Marqui (2007a) with the *lagged coherence* (ρ^2^), i.e.,

$$\begin{array}{l} \rho_{ij}^{2}\left(f\right):=\frac{{\left[ℑ\left(S_{ij}\left(f\right)\right)\right]}^{2}}{S_{ii}\left(f\right)\;S_{jj}\left(f\right)-{\left[ℜ\left(S_{ij}\left(f\right)\right)\right]}^{2}}, \end{array}$$

where the instantaneous, i.e., zero lag, contributions are partialled out. This formulation is equivalent to the “*corrected” imaginary coherence* defined in Ewald et al. (2012).

Based on similar arguments, the *imaginary part of PLV* (iPLV; Palva and Palva, 2012), i.e.,

$$\begin{array}{l} \text{iPL}{\text{V}}_{\text{ij}}\left(f\right):=\left|\langle ℑ\left(e^{\iota \;{\Delta \theta }_{ij}\left(f\right)}\right)\rangle \right| \end{array}$$

solely detects phase-lagged coupling.

Instead of averaging the imaginary part of $e^{\iota \;{\Delta \theta }_{ij}\left(f\right)}$, the *phase-lag-index* (PLI; Stam et al., 2007) quantifies the asymmetry in the distribution of the phase difference across realizations, when this distribution is centered around zero, as

$$\begin{array}{l} \text{PL}{\text{I}}_{\text{ij}}\left(f\right):=\left|\langle sign\left({\Delta \theta }_{ij}\left(f\right)\right)\rangle \right|. \end{array}$$

To improve the robustness of PLI with respect to correlated and uncorrelated noise, as well as to increase the statistical power of the metric, Vinck et al. (2011) proposed the *weighted PLI* (wPLI) as a weighted average of the signs of the phase difference, i.e.

$$\begin{array}{l} \text{wPL}{\text{I}}_{\text{ij}}\left(f\right):=\frac{\left|\langle |ℑ\left(X_{i}\left(f\right)\;X_{j}^{*}(f)\right)|sign\left({\Delta \theta }_{ij}\left(f\right)\right)\rangle \right|}{\langle \left|ℑ\left(X_{i}\left(f\right)\;X_{j}^{*}(f)\right)\right|\rangle }\;, \end{array}$$

where the weights are equal to the imaginary part of the cross-spectra computed within realizations.

**Figure 4:**
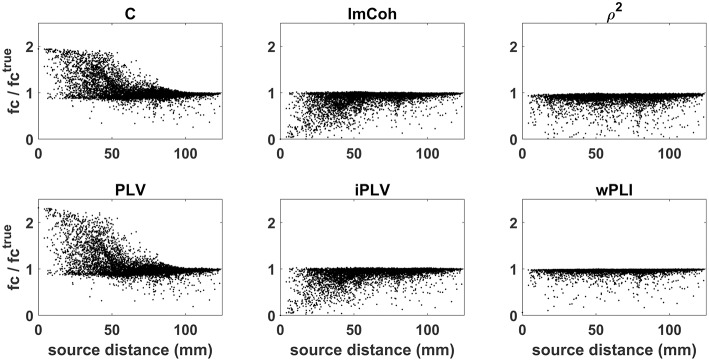
Effects of source leakage on different connectivity measures. Functional connectivity was estimated by using: coherence (C; top left), phase locking value (PLV; bottom left), imaginary part of coherency (ImCoh; top center), imaginary part of phase locking value (iPLV; bottom center), lagged coherence (ρ^2^; top right), weighted phase lag index (wPLI; bottom right). For each connectivity measure, the ratio between the estimated functional connectivity values and the true value is plotted as a function of the distance between sources 1 and 2. Artificially inflated connectivity estimates (ratio values larger than 1) can be observed for coherence and phase locking value, but not for other measures which were specifically designed to handle the effects of source leakage.

The effects of source leakage on these different connectivity measures is shown in Figure 4. Synthetic source time series were simulated as described in section Source space MEG. Functional connectivity between reconstructed source time courses was estimated by using either coherence (C; top left), or phase locking value (PLV; bottom left), or imaginary part of coherency (ImCoh; top center), or imaginary part of phase locking value (iPLV; bottom center), or lagged coherence (ρ^2^; top right), or weighted phase lag index (wPLI; bottom right). For each connectivity measure, the ratio between the estimated functional connectivity values and the true value (i.e., the one obtained directly from the generated source time sources) is plotted as a function of the distance between sources 1 and 2. Artificially inflated connectivity estimates (ratio values larger than 1) can be observed for coherence and phase locking value as an effect of source leakage. Of note, this effect is not limited to close by sources but is present also when the sources are far apart (e.g., with distance of about 6 cm). Conversely, measures which were specifically designed to handle the effects of source leakage (e.g., ImCoh or iPLV) are rather conservative (ratio values smaller than 1) and tend to underestimate true connectivity values.

The following two methods are conceived to investigate the directionality of phase coupling. Specifically, Nolte et al. (2008) exploited the properties of the complex-valued coherency, and introduced the *phase slope index* (PSI) as

$$\begin{array}{l} \;{\text{PSI}}_{ij}\left(F\right):=\sum_{f\in F}ℑ\left(C_{ij}\left(f+df\right){\left(C_{ij}\left(f\right)\right)}^{*}\right)\\ =\sum_{f\in F}|C_{ij}\left(f+df\right)\;C_{ij}^{*}\left(f\right)|\sin\left(\Delta \theta_{\text{ij}}\left(\text{f}+\text{df}\right)-\Delta \theta_{\text{ij}}\left(\text{f}\right)\right), \end{array}$$

where *df* is an incremental step in the frequency domain, and *F* denotes the frequency band of interest. PSI is essentially a weighted average of the slope of the phase difference Δθ_*ij*_(*f* + *df*) − Δθ_*ij*_(*f*) across *F*, whose sign provides a measure of the directionality. In particular, a positive value indicates that the signal *i* precedes (thus leads) the signal *j*, while a negative value indicates the opposite (Basti et al., 2017). Similarly to ImCoh, PSI requires a phase slope between signals to be non-vanishing, and thus it does not lead to artifactual detection of directed connectivity due to artificial zero-lag correlations.

Lobier et al. (2014) applied the concept of transfer entropy (Schreiber, 2000), developed in the framework of information theory (Shannon, 1948), to the assessment of directed phase coupling, by introducing the *phase transfer entropy* (pTE). The pTE from a signal *i* to a signal *j* is defined as

$$\begin{array}{l} pTE_{ij}\left(F,\tau \right):=H\left(\theta_{j}\left(F,t\right),\theta_{j}\left(F,t-\tau \right)\right)+H(\theta_{j}(F,t\\ -\tau ),\theta_{i}\left(F,t-\tau \right))-H\left(\theta_{j}\left(F,t-\tau \right)\right)\\ -H\left(\theta_{j}\left(F,t\right),\;\theta_{j}\left(F,t-\tau \right),\theta_{i}\left(F,t-\tau \right)\right). \end{array}$$

In the above notation, θ_*j*_(*F, t*) denotes the instantaneous phase signal obtained from, e.g., Hilbert or wavelet transform of the bandpass filtered signal *j*; *F* denotes the frequency band of interest; τ denotes a given time delay; and *H*(*Z*) : = −∫*p*(*Z*) ln (*p*(*Z*)) denotes the information entropy of the random variable *Z*, where *p*(*Z*) is the probability density function (see Lobier et al., 2014, and references therein for more details). The direction of the phase coupling can be assessed through the comparison between the pTE from signal *i* to *j* and the one from *j* to *i* (Hillebrand et al., 2016). pTE has the advantage over the traditional transfer entropy measure of being able to handle the artificial zero-lag correlation effects (Lobier et al., 2014).

The above described methods are univariate methods since they are designed to assess connectivity between pairs of univariate signals, e.g., MEG signals at sensor level. When dealing with brain activity estimated from MEG signals, e.g., according to one of the strategies described in section Source space MEG, the most general situation results into three brain activity components (also termed dipole orientations or dipole moments) at each location of the discretized brain. To apply univariate connectivity methods on these three-dimensional activity components, it is necessary to perform a dimensionality reduction to one-dimensional signals which might result in a loss of information and in a subsequent inaccuracy in the estimation of connectivity. Conversely, multivariate connectivity methods are specifically designed to take into account the whole information contained in the multidimensional nature of the input signals, i.e., they do not require a dimensionality reduction of the input signals. In the following subparagraph, a review of multivariate methods for MEG connectivity is provided.

#### Multivariate Methods

Let $x_{I}\left(t\right)={\left(x_{i_{1}}\left(t\right),\ldots ,x_{i_{N}}\left(t\right)\right)}^{\text{T}}$ and $x_{J}\left(t\right)={\left(x_{j_{1}}\left(t\right),\ldots ,x_{j_{M}}\left(t\right)\right)}^{\text{T}}$ be the multivariate time series for the *N*-dimensional signals *I* and the *M*-dimensional signals *J*, where *T* denotes the transpose operator. For instance, these could represent the three-dimensional vector-source activities at two given brain locations, and thus they would have a dimension *N* = *M* = 3, or they could indicate two different multivariate time series consisting of the activities of all the sources within a region of interest, and thus they would have a dimension equal to the number of locations belonging to those parcels. Let also *X*_*I*_(*f*) and *X*_*J*_(*f*) be the corresponding vector Fourier transforms. The cross-spectral density matrix between *x*_*I*_(*t*) and *x*_*J*_(*t*) at a given frequency *f* is defined as

$$\begin{array}{l} S_{IJ}\left(f\right)\langle X_{I}\left(f\right)\;{\text{X}}_{J}^{H}\left(f\right)\rangle :\text{​}=\\ \;\left[\begin{array}{ccc} \langle X_{i_{1}}\left(f\right)\;X_{j_{1}}^{*}\left(f\right)\rangle & \cdots & \langle X_{i_{1}}\left(f\right)\;X_{j_{M}}^{*}\left(f\right)\rangle \\ \vdots & \ddots & \vdots \\ \langle X_{i_{N}}\left(f\right)\;X_{j_{1}}^{*}\left(f\right)\rangle & \cdots & \langle X_{i_{N}}\left(f\right)\;X_{j_{M}}^{*}\left(f\right)\rangle \end{array}\right], \end{array}$$

where (∙)^H^ denotes the Hermitian conjugate of a matrix. The elements of **S**_*IJ*_(*f*) are the cross-spectra between all the pairwise combinations of univariate time series of *I* and *J*.

The *multivariate interaction measure* (MIM, Ewald et al., 2012) is an index of the total phase coupling between vector-signals *I* and *J*, and it is defined as

$$\begin{array}{l} {\text{MIM}}_{IJ}\left(f\right):=Tr\left({\left(S_{II}^{ℜ}\left(f\right)\right)}^{-1}\;S_{IJ}^{ℑ}\left(f\right)\;{\left(S_{JJ}^{ℜ}\left(f\right)\right)}^{-1}{\left(S_{IJ}^{ℑ}\left(f\right)\right)}^{\text{T}}\right), \end{array}$$

where the superscripts ℜ and ℑ denote the real and imaginary part of the complex-valued cross-spectral density matrices, (∙)^−1^ and *Tr*(∙) are the inverse and trace operators. MIM is the generalization of ImCoh to multivariate time series analysis and, in the case of two univariate time series, it coincides with the squared ImCoh. Similarly to ImCoh, MIM does not lead to artifactual phase coupling detections due to artificial zero-lag correlations. Furthermore, MIM is invariant under invertible and static linear transformations of *x*_*I*_(*t*) and *x*_*J*_(*t*) (Ewald et al., 2012), such as rotations of the physical coordinate system in which the MEG source space is defined.

The generalization of the lagged coherence to the multivariate case will be here referred to as the *multivariate lagged coherence* (*P*; Pascual-Marqui, 2007b), and it has the form

(f)IJ:​=lndet(ℜ[SJJ(f)SJI(f)SIJ(f)SII(f)])/det(ℜ[SJJ(f)00TSII(f)])det([SJJ(f)SJI(f)SIJ(f)SII(f)]) / det([SJJ(f)00TSII(f)])

where det(∙) denotes the determinant of a matrix, and **0** is a *M* × *N* matrix of zeros. In the particular case of univariate time series, the following identity holds: $P_{ij}\left(f\right)=-\ln\left(1-\rho_{ij}^{2}\left(f\right)\right)$. Similarly to MIM, the multivariate lagged coherence is invariant under invertible and static linear transformations of input signals.

To assess the directionality from pairs of vector-signals, Basti et al. (2018) generalized the definition of PSI to multivariate time series, called *multivariate phase slope index* (MPSI). MPSI is defined as

$$\begin{array}{l} {\text{MPSI}}_{\text{IJ}}(\text{F}):=4\sum_{f\in F}\text{Tr}({\left(S_{II}^{ℜ}(\text{d}f+f)+S_{II}^{ℜ}(f)\right)}^{-1}\\ \;S_{IJ}^{ℑ}(\text{d}f+f)\;{\left(S_{JJ}^{ℜ}(\text{d}f+f)+S_{JJ}^{ℜ}(f)\right)}^{-1}S_{JI}^{ℜ}(f)\\ \;+{\left(S_{II}^{ℜ}\left(\text{d}f+f\right)+S_{II}^{ℜ}\left(f\right)\right)}^{-1}\;S_{IJ}^{ℜ}\left(\text{d}f+f\right)\\ \;{\left(S_{JJ}^{ℜ}(\text{d}f+f)+S_{JJ}^{ℜ}(f)\right)}^{-1}S_{JI}^{ℑ}\left(f\right)) \end{array}$$

where *df* and *F* denote an incremental step in the frequency domain and the frequency band of interest, respectively. MPSI solely detects the directionality of phase-lagged coupling; a positive value of MPSI indicates that the vector-signal *I* leads the vector-signal *J*, while a negative value indicates the opposite. Moreover, similarly to MIM, MPSI is invariant under invertible and static linear transformations, and thus it is independent on rotations of the physical coordinate system of MEG source space (Basti et al., 2018). In the case of two univariate time series, MPSI coincides with PSI, apart from a normalization factor.

### Cross-Frequency Phase Coupling Methods

In section Frequency-specific phase coupling methods, we introduced a large set of connectivity methods based on phase coupling of oscillatory signals at the same frequency, which has been hypothesized as a mechanism for communication, at large scale for slower rhythms and at small scale for higher frequencies. Additionally, neuronal oscillations at different frequencies can also couple according to different possible relations between their phases, their amplitudes or in a phase-to-amplitude mode (Jensen and Colgin, 2007). This cross-frequency coupling has been hypothesized as a mechanism for information integration across different spatial scales characteristic of the faster and slower oscillations.

In this review, consistently with what already presented for coupling at the same frequency, we focus on methods to detect phase to phase cross-frequency coupling.

A popular cross-frequency measure in this framework is the *n:m synchronization index* (Rosenblum et al., 1996; Tass et al., 1998). This measure relies on the estimation of the generalized phase difference $\Phi_{ij}^{n:m}$ between two signals *i* and *j*, i.e.,

$$\begin{array}{l} \Phi_{ij}^{n:m}\left(f_{1},f_{2},t\right):=n\;\theta_{i}\left(f_{1},t\right)-m\;\theta_{j}\left(f_{2},t\right), \end{array}$$

where *f*_1_and *f*_2_ are two frequencies whose ratio is given by the integers *n* and *m*, i.e., *n f*_1_ = *m f*_2_, and θ_*i*_(*f, t*) denotes the instantaneous phase signal. A *n*:*m* coupling between two signals occurs when the mean resultant length of $\Phi_{ij}^{n,m}$ is non-vanishing (Tass et al., 1998). This can be evaluated, e.g., through the *n*:*m phase-locking-value* (PLV^*n*:*m*^) which is defined as

$$\begin{array}{l} {\text{PLV}}_{i,j}^{n:m}\left(f_{1},f_{2}\right):=\left|\langle e^{\iota \;\Phi_{ij}^{n,m}\left(f_{1},f_{2},t\right)}\rangle \right|. \end{array}$$

where 〈∙〉 denotes the average over signal realizations and time (Palva et al., 2005; Siebenhühner et al., 2016).

A different class of cross-frequency phase-coupling measures relies on the estimation of the third order spectrum, or cross-bispectrum, between signals (Nikias and Petropulu, 1993). The most general expression for the cross-bispectrum involves three signals, *i*, *j* and *k*, and three frequencies, *f*_1_, *f*_2_ and *f*_3_ = *f*_1_ + *f*_2_, and it has the form

$$B_{ijk}\left(f_{1},f_{2}\right):=\langle X_{i}\left(f_{1}\right)\;X_{j}\left(f_{2}\right)\;X_{k}^{*}(f_{1}+f_{2})\rangle .$$

The frequency *f*_3_ is set to *f*_1_ + *f*_2_ because all other choices lead to vanishing cross-bispectra under stationarity assumption (Chella et al., 2016b; Shahbazi Avarvand et al., 2018). A pairwise bispectral analysis between two signals can be accomplished by setting two out of the three signal indices equal to each other, e.g., *i* = *j* which yields *B*_*ik*_ (*f*_1_, *f*_2_) *B*_*iik*_ (*f*_1_, *f*_2_).

The *bicoherency* (Nikias and Petropulu, 1993) is the normalized version of the cross-bispectrum, which is the analogous of the coherency for the cross-spectrum. The absolute value of bicoherency, i.e., the bicoherence, is a measure of the coupling between the phases in signals *i* and *j* at two possibly different frequencies, θ_*i*_(*f*_1_) and θ_*j*_(*f*_2_), with respect to the phase in signal *k* at a third frequency which is the sum of the other two, θ_*k*_(*f*_1_ + *f*_2_), such that the mean resultant length of the phase difference $\Phi_{ij}^{q}\left(f_{1},f_{2}\right)\theta_{i}\left(f_{1}\right)+\theta_{j}\left(f_{2}\right)-\theta_{k}\left(f_{1}+f_{2}\right)$ is non-vanishing. Such a phenomenon is called quadratic phase coupling, and it conceptually different from the *n:m* coupling described above. There is one case in which the two phenomena coincide, that is the case of *f*_1_ = *f*_2_ =:*f* and *f*_3_ = 2*f*, in which the quadratic phase coupling involves only two frequency components, i.e., one frequency and its double, thus matching the 1:2 phase locking.

Similarly to coherence, bicoherence estimates are inflated by artificial zero-lag correlations. To face this issue, Chella et al. (2014, 2016b) proposed to use the *antisymmetric part of bicoherency*,

$$\begin{array}{l} {\text{aBic}}_{ijk}\left(f_{1},f_{2}\right)\\ \;:=\frac{B_{ijk}\left(f_{1},f_{2}\right)-B_{kji}\left(f_{1},f_{2}\right)}{Q_{i}\left(f_{1}\right)\;Q_{j}\left(f_{2}\right)\;Q_{k}\left(f_{1}+f_{2}\right)+Q_{k}\left(f_{1}\right)\;Q_{j}\left(f_{2}\right)\;Q_{i}\left(f_{1}+f_{2}\right)} \end{array}$$

where $Q_{i}\left(f\right)=\sqrt[{3}]{\langle |X_{i}\left(f\right){|}^{3}\rangle }$ (Shahbazi et al., 2014). The aBic is the difference between two bicoherencies where two signal indices have been switched, i.e., *i* and *k* in the above equation.

## Exemplary Applications of Phase Coupling to Assess Brain Connectivity

In the following, exemplary applications of phase coupling estimated from MEG data to neuroscience are recalled. To this end, we will focus on three widely studied domains in which phase coupling methods have contributed in disclosing the putative mechanisms underlying brain operations: resting state, visuospatial attention, and working memory. The aim of this chapter is not to give an exhaustive review of respective literature but rather to give the reader an idea of how phase coupling methods from MEG data has contributed in the study of the covered fields. It is worth noting that the same domains that we recall here, have been studied also with EEG and the contribution of these studies to our knowledge in the respective domains cannot be underestimated (e.g., Crespo-Garcia et al., 2013, Sauseng et al., 2004, 2005a,b). However, in line with the perspective of this review we will focus on the studies that have used phase coupling methods on source-space MEG data. Finally, it needs to be highlighted that applications of the phase coupling methods are not limited to the covered examples. Specifically, the value of neurophysiological signals as potential biomarkers has been recognized. Recent literature have identified the potential in using neurophysiological signals for diagnostic purposes in schizophrenia (Bowyer et al., 2015), autism spectrum disorders (Port et al., 2015), post-traumatic stress disorder, mild traumatic brain injury (Huang et al., 2016), Alzheimer's disease (Maestú et al., 2019) and epilepsy (Soriano et al., 2017). Furthermore, the use of MEG-based markers is not limited to diagnostics but shows promise also in probing cognitive decline in multiple sclerosis (Schoonhoven et al., 2018) and even in treatment response monitoring (Light and Swerdlow, 2015) and drug development (Javitt et al., 2008).

### MEG Resting State Connectivity

Resting state is defined as an intrinsic state in which the subject is not engaged in a specific task or acting in response to a given stimulus. More specifically, according to Snyder and Raichle (2012), “… *rest* is an operational definition referring to a constant condition without imposed stimuli or other behaviorally salient events.” The definition of resting state encompasses eyes closed or eyes open conditions, the latter with or without visual fixation.

In the first decade of the 2000s, the study of the functional architecture of the resting brain has become an emerging line of research in neuroscience, although it poses some challenges (e.g., signal-to-noise ratio and lack of temporal reference) due to rest being a quite uncontrolled experimental paradigm compared to task-based experiments. Specifically, a growing number of fMRI studies have investigated functional topographies in the resting brain, as well as the relation of these topographies to evoked-response patterns and to anatomy (for a review see Fox and Raichle, 2007). Taken together, these studies have shown that the resting brain is characterized by distributed large-scale cortical networks of coherent activity, namely RSNs, which cover at least the 66% of the human brain (Deco and Corbetta, 2011) and account for the largest part of brain energy consumption (Raichle, 2006). The RSNs most commonly identified by fMRI include (Yeo et al., 2011): The Default Mode Network (DMN), the Dorsal Attention Network (DAN), the Ventral Attention Network (VAN), the Sensori-Motor Network (SMN), the Visual Network (VN), the Fronto-Parietal Control Network (FPN), the Language Network (LN).

**Figure 5:**
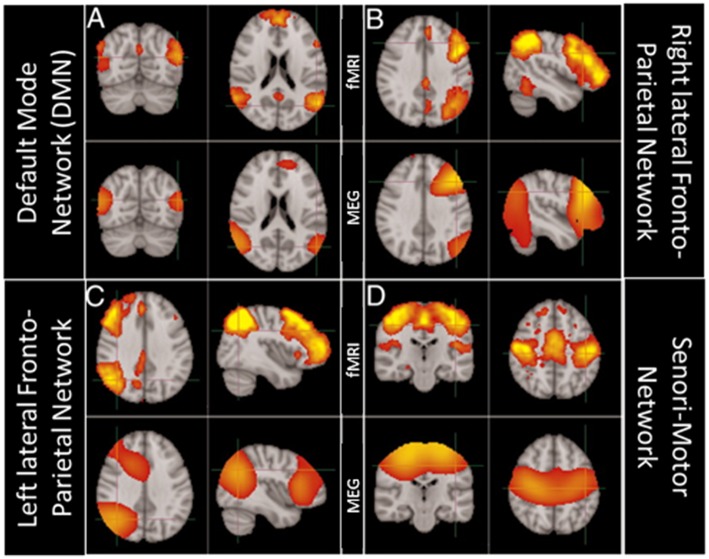
Comparison of brain networks obtained using ICA independently on MEG and fMRI data. Modified from Brookes et al. (2011b) (CCBY license). **(A)** Top row: fMRI derived Default Mode Network (DMN); bottom row MEG derived DMN. **(B)** Top row: fMRI derived Right lateral FrontoParietal Network; bottom row MEG derived Right lateral FrontoParietal Network. **(C)** Top row: fMRI derived Left lateral FrontoParietal Network; bottom row MEG derived Left lateral FrontoParietal Network. **(D)** Top row: fMRI derived Sensori-Motor Network; bottom row MEG derived Sensori-Motor Network.

Nevertheless, given that fMRI BOLD activity is not a direct measure of neuronal activity, neuroscientists have become more and more interested in using electrophysiological techniques, as a stand alone or in combination with fMRI, to unravel the electrophysiological correlates of RSNs and their potential relation to brain oscillations (see e.g., Mantini et al., 2007). In the last decade, advancements in MEG data analysis have made it possible to rely on source space MEG connectivity to study RSNs. The richness of the MEG signal has offered several perspectives to look at RSNs (Larson-Prior et al., 2013) and, from that, to enhance the understanding of the putative mechanisms underlying their formation. The first studies (de Pasquale et al., 2010, 2012) used a signal analysis strategy aimed at extracting slow fluctuations of the MEG signal and investigating their temporal correlation, closely resembling the signal analysis strategy used for resting state fMRI data. Additionally, spatial Independent Component Analysis (ICA) has been used on resting state MEG (rsMEG) data to extract networks of coherent fluctuations, showing that MEG RSNs feature significant similarity in their spatial structure compared with fMRI RSNs as evidenced in Figure 5 (Brookes et al., 2011b).

**Figure 6:**
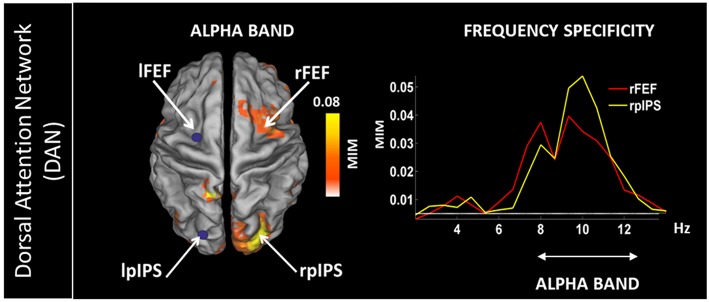
**(Left)** Topography of the phase synchronization between the major nodes of the Dorsal Attention Network (DAN) in the left hemisphere, i.e., left Frontal Eye Field (lFEF), left posterior Inferior Parietal Sulcus (lpIPS) and the homologous DAN nodes in the alpha band (right FEF and right pIPS). **(Right)** Frequency specificity of the coupling shown on the left. Modified from Marzetti et al. (2013).

In addition, rsMEG has shown its potential to move beyond fMRI RSNs by relying on connectivity metrics which can exploit the rich frequency content of the MEG signal (typically 1–80 Hz; Baillet, 2017) by assessing phase locking between oscillatory activities. Metrics based on phase coupling have thus been used to assess frequency specific patterns of coupled activity. By using Multivariate Interaction Measure (Ewald et al., 2012), our group has shown that coupling within and between RSNs features frequency specific signatures (Marzetti et al., 2013). Specifically, by seeding parietal and frontal node of the Dorsal Attention Network in the left hemisphere (namely left inferior parietal sulcus and frontal eye field) we observed coupling with the contralateral regions specific to the delta and the alpha frequency bands (Figure 6 for the alpha band). Concurrently, we observed that the same DAN nodes are coupled to nodes belonging to other networks in different specific frequencies: DAN and SMN couple in the beta band while DAN and visual network couple in the alpha band. Overall, our work demonstrated that brain networks that can be observed with resting state MEG show frequency specificity and that the involved frequencies overlap to those observed during tasks which are known to recruit regions in these networks.

**Figure 7:**
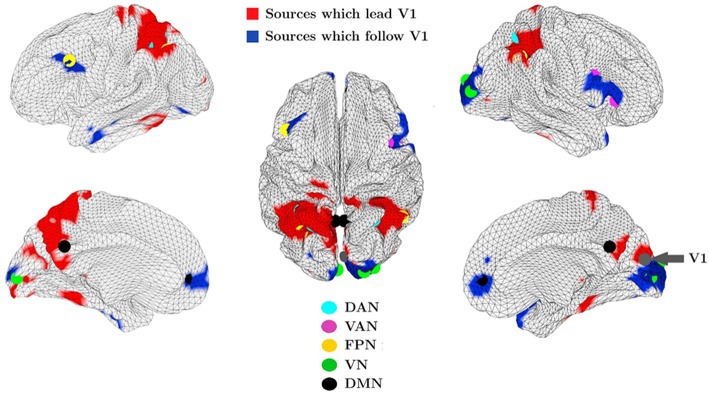
Group averaged map in the alpha band between the primary visual cortex (V1) and all other locations over the cortex obtained by MPSI. The cortical locations in red exert an influence on V1 while V1 exerts an influence on the regions in blue. The dots, in color according to the legend, represent resting state network nodes which overlap areas which lead or follow V1. Modified from Basti et al. (2018) (CCBY license).

More recently, our group introduced a novel phase coherence metric, namely the Multivariate Phase Slope Index, to investigate the directionality of functional coupling in MEG and EEG data (Basti et al., 2018). This novel metric has been used to investigate the directionality of functional coupling in the alpha frequency band between the visual network and the whole brain in resting state MEG data from a large cohort of healthy subjects from the Human Connectome Project (Larson-Prior et al., 2013). With this approach, we observed sets of regions belonging to various RSNs which either lead or follow the primary visual cortex (Basti et al., 2018) as shown in Figure 7. For example, we observed coupling between the primary visual cortex (V1) and the Dorsal Attention Network. Parietal areas of the latter lead V1, in line with the notion of a feedback mechanism, while no clear evidence of follower or leader relationship was observed between V1 and the frontal regions in the DAN, suggesting that coupling of V1 to frontal DAN areas observed in Marzetti et al. (2013) can possibly exhibit both feed-forward and feedback characteristics which might lead to the absence of a clear directionality observed in Basti et al. (2018).

Additionally, with mPSI we observed a consistent relationship in the alpha band between V1 and the dorsolateral pre-frontal cortex (dlPFC) with the latter following V1 in accordance with the putative role of dlPFC in action preparation in response to the visual stimulus (Heekeren et al., 2006). For the interested reader, we refer to the original paper for details on the method and on the coupling of V1 to the other RSNs shown in Figure 7.

While literature on resting state frequency specific coupling by MEG is relatively abundant, less has been investigated about cross-frequency coupled networks. To date, few studies have investigated the coupling between the local phase of a low frequency oscillation and the local amplitude of a high frequency oscillation, i.e., Phase-Amplitude Coupling (PAC). In their interesting paper, Roux et al. (2013) showed that in resting-state MEG, local gamma-band activity is coupled with the phase of the alpha band rhythm in the visual cortex. Furthermore, Florin and Baillet (2015) showed that resting-state networks derived from signal model based on the PAC, substantially overlapped with DMN, DAN/SMN, VN, and right FPN, indicating that the PAC is an important component of the network generation in the resting-state. More recently, an effort to disclose n:m-cross-frequency phase coupling RSNs and to disentangle these networks from phase-amplitude coupling networks has been made by Siebenhühner et al. (2019). Nevertheless, the topic still needs further investigation.

### Visuospatial Attention

As the selection of information relevant to behave in the environment is a fundamental process, the human brain is endowed with selective attention mechanisms necessary to route information in order to perform different tasks while ignoring distracting input. MEG and EEG evidence show that long range coupling of neuronal oscillations through phase coherence in a large-scale functional connectivity network, identified through the synchronization of neuronal oscillations, subserve this attentional selection process (Siegel et al., 2008; Doesburg et al., 2009; Sauseng et al., 2005b; Lobier et al., 2018; D'Andrea et al., 2019).

**Figure 8:**
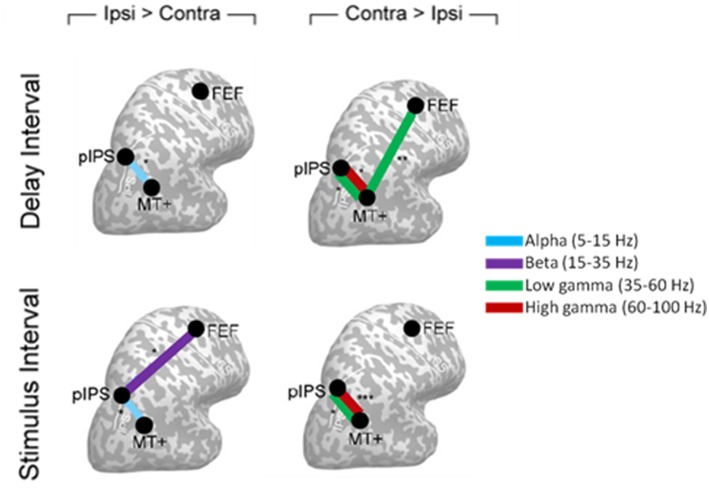
Cartoon of the interregional phase coherence modulated by attention in Siegel et al. (2008), displayed on a flattened cortex. Circles indicate cortical areas in which phase coupling was observed to be modulated, namely: posterior intraparietal sulcus (pIPS), middle temporal (MT+), and frontal eye field (FEF). Panels on the left show the cortical areas between which attention significantly reduced phase coherence in the hemisphere ipsilateral as compared to contralateral to the attended hemifield. Panels on the right highlight the corresponding attentional enhancement of phase coherence. The different frequency bands (alpha, beta, low gamma, and high gamma) involved are indicated by different colors.

The study from Siegel et al. (2008) provided one of the first evidence for a modulation of phase coherence induced by attention with different spectral signatures and stimulus dependence between cortical areas. Indeed, Siegel et al. recorded MEG data in eight subjects performing a motion discrimination task, in which they had to discriminate the motion direction of a cloud of dots in the cued hemifield ignoring the uncued hemifield. Coupling between five regions specifically implicated in the attention mechanisms and visual motion processing (see Figure 8 for details on the regions) was estimated through phase coherence, either in the stimulus or in the delay interval. A relative enhancement of phase coherence was found at high frequencies (35–100 Hz) and a relative decrease was found at low frequencies (5–35 Hz) in the hemisphere contralateral to the attended hemifield (Figure 8). Specifically, in the delay interval a reduction of gamma band connectivity was observed in the hemisphere ipsilateral to the cued hemifield between the posterior Intra Parietal Sulcus (pIPS) and middle Temporal (MT+), sustained also during the stimulus presentation, and between FEF and MT+. This reduction was paired to an enhancement of alpha phase coherence between pIPS and MT+ in the all intervals (Delay and Stimulus) as well as to an enhancement of phase coherence in the beta band between FEF and MT+ specific to the Stimulus Interval. By using coherence it was shown that attentional modulations of synchronization between ROIs is spatially selective, across all bands and intervals, representing a long range synchronization that does not depend on power effects.

More recently, another study (Lobier et al., 2018) investigated the role of long-range phase coupling in visuospatial attention processes, without a priori selection of frequency-bands or regions of interest. This study is particularly relevant from a methodological point of view since here phase coupling was assessed both using the weighted Phase Lag Index and the Phase Locking Value, the former to minimize the contribution of source leakage and the latter to exclude changes in phase lags without a change in coupling strength. Cortical activity was recorded with MEG from fourteen healthy participants performing a cued stimulus discrimination task. After a rightward or leftward cue presentation, subjects had to discriminate between two geometrical shapes with two different contrast conditions. The shapes were presented in the attended or non-attended hemifield. The results showed an increased inter-areal phase coupling in the high-alpha frequency band between visual, parietal, and frontal cortices. Interestingly, no major difference was observed between phase coupling assessed through wPLI or through PLV indicating that the observed findings cannot be attributed merely to source leakage nor to “systematic changes in phase lags without a change in coupling strength” (Lobier et al., 2018). No other frequency bands such as gamma and beta were found to be involved in this task.

A recent study from our group, D'Andrea et al. (2019), further investigated, through MIM, PSI and aBic, the frequency specificity of the inter-areal phase coupling induced by attention, and, at the same time, the relationship between this inter-areal phase coupling and anatomical connections. Magnetoencephalographic data from 28 participants performing a visuospatial attention task and high angular resolution diffusion imaging (HARDI) magnetic resonance (MR) data, in the same subjects, were used. From MEG data seed-based MIM in the alpha and beta frequency bands, and cross-frequency aBic coupling between these two frequency ranges were quantified. From MR data, the Superior Longitudinal Fasciculus branches were reconstructed and asymmetry for each branch was calculated as the difference between the volume of the left branch and that of the right branch (for details see D'Andrea et al., 2019).

**Figure 9:**
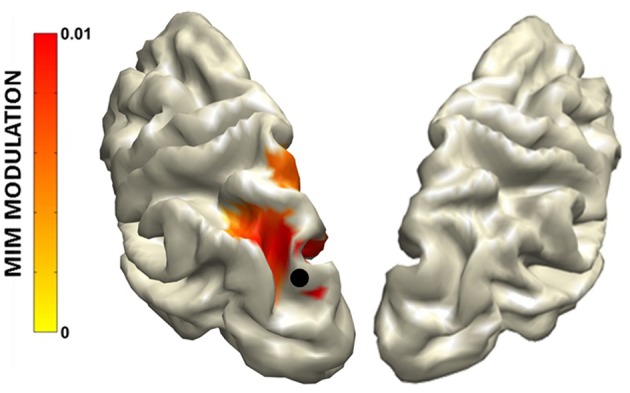
Grand average MIM functional connectivity modulation (mMIM) with respect to the left superior occipital cortex (indicated by the black dot) for the “Attend Left” condition vs. Baseline in the alpha band. Modified from D'Andrea et al. (2019) (CCBY license).

The MIM and the PSI highlighted an increase of functional connectivity in the alpha band from parietal areas to the occipital cortex (Figure 9) always larger in the hemisphere ipsilateral to the hemifield to which attention is directed.

**Figure 10:**
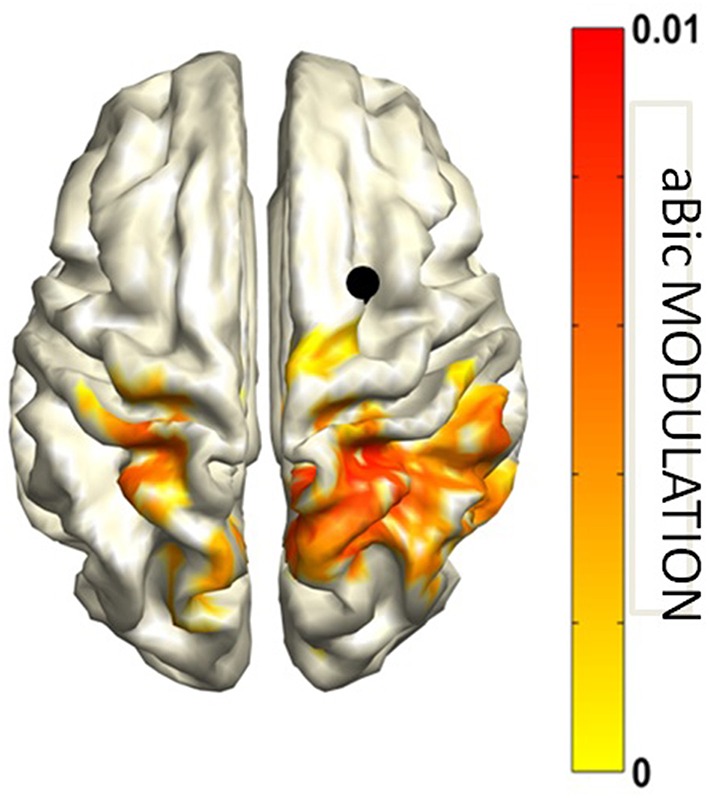
Attend Left and Attend Right conjunction map of aBic modulation vs. Baseline in the alpha-beta band with respect to the right frontal cortex seed (indicated by the black dot). Modified from D'Andrea et al. (2019) (CCBY license).

This interaction, reflecting a top down mechanism exerted by parietal areas for the inhibition of visual cortices, was found to be related to behavioral performance and to indices of anatomical connectivity. Specifically, the hemispheric asymmetry of this occipito-parietal modulation of connectivity was predicted by the asymmetry in the volume of the first and the second tracts of the Superior Longitudinal Fasciculus and was positively correlated to the accuracy in performing the task. Additionally, the antisymmetric part of bicoherency revealed an increase in alpha-beta coupling, induced by the cue presentation, between the right FEF and parietal areas in both hemifields (Figure 10). No other frequency band was observed.

Overall, these results highlight that neuronal oscillations and functional connectivity at low frequencies are strongly implicated in attentional mechanisms and are possibly related to functional inhibition through feedback mechanisms (Jensen and Mazaheri, 2010). Nevertheless, further investigation is needed to clarify the specific role of the different frequency (sub-)bands observed in long-range phase coupling. Indeed, while it is conceivable that gamma frequencies are implicated in bottom-up information processing, still poor evidence demonstrate the involvement of long-range gamma-band coherence in top-down processing (Siegel et al., 2008; Gregoriou et al., 2009) and further investigation are needed to associate different frequency bands with specific mechanism of feedback and feed-forward interactions (Siegel et al., 2012).

### Working Memory

Complex cognitive tasks such as language comprehension, reasoning and learning rely on our brains' ability to temporary maintain and manipulate information, that is, working memory (WM) (Baddeley, 1992). The phase coupling is fundamental neural mechanism that supports neural communication and it is therefore an apparent prospect for the underlying neural mechanism of the WM. Indeed, the role of phase coupling in memory processes has been studied extensively and the literature supports the relevance of phase coupling in memory processes (see Fell and Axmacher, 2011 for review). The majority of the studies examining the phase coupling in WM have relied on EEG data. Moreover, most of the studies have evaluated the phase coupling in sensor space, which, as discussed earlier in this review, is problematic. Here we focus on those WM studies that have examined the role of phase coupling in source space using MEG data, with the benefit of being more specific about the involved brain areas.

Palva et al. (2010) used combined recordings of MEG and EEG to characterize phase coupled networks underlying the maintenance phase of delayed match to sample task (DMS). The inter-areal phase coupling was assessed by PLV, and statistically significant couplings were represented as undirected graphs in which vertices were the cortical areas and edges the significant couplings. Network hubs were identified by using vertex degree and betweenness centrality (graph theoretical indices). The phase coupling in alpha, beta, and gamma bands was shown to be sustained and stable throughout the visual working memory (VWM) retention period. Increasing memory load strengthened interareal coupling among the frontoparietal regions particularly strongly in the alpha band, while beta and gamma bands underlay coupling in visual regions. These observations, combined with the trend of alpha band hubs being located more frontally compared to beta and gamma band hubs, led to the suggestion that alpha band network underlies higher level attentional functions. Conversely, coupling in the beta and gamma bands were suggested to be involved in binding neuronal representations. The analysis of the most important hubs revealed that the intraparietal sulcus (IPS) was the most central hub predicting individual WM capacity and it also connected the visual and frontoparietal networks (beta and gamma). Therefore, it was concluded that IPS might control the information flow between visual representation and frontoparietal attention network (Palva et al., 2010).

Another study examined phase coupling (wPLI) networks underlying the maintenance phase of DMS task in 6-year old children (Sato et al., 2018). The results showed that, compared to baseline, whole brain connectivity in the alpha band was increased during the retention period. Sato et al. (2018) associated the alpha band connectivity to attentional functions similarly to Palva et al. (2010). The whole brain connectivity was significantly greater in correct trials compared to incorrect trials. The authors speculated that the decreased connectivity associated with incorrect trials might be due to children's inability to dedicate enough attentional resources to trials which were incorrectly answered. The children also showed more fronto-temporal coupling rather than the more common fronto-parietal coupling. Furthermore, key network hubs were located to the left inferior frontal triangularis, left hippocampus, left middle temporal gyrus, and the left superior temporal gyrus. It was stated that these results might indicate that the children used rehearsal strategies and recognition processes to perform the task. Further evidence of fronto-temporal coupling during the maintenance phase of WM task was presented in a study by Daume et al. (2017). They combined directional and non-directional metrics to show that phase coupling in theta/alpha range (ImCoh) was significantly greater in memory condition compared to control and the direction of this coupling (PSI) was from frontopolar cortex to inferior temporal cortex, possibly indicating that the frontopolar cortex to temporal area coupling is involved in coordinating the maintenance of visual object representations in a top down manner (Daume et al., 2017).

Alpha band phase coupling has also been related to inhibitory process in the context of WM. Overall alpha band connectivity (wPLI) was found to be stronger during the retention period of a variant of Sternberg task compared to baseline (Wianda and Ross, 2019). The most prominent connections involved occipital, frontal, and mid-brain sources. Long-range connectivity was observed between visual cortices and the retrosplenial cingulate cortex and thalamic sources. Furthermore, pre-frontal cortices were connected to midbrain sources and temporal cortices. The authors proposed that alpha coupling between frontotemporal and occipital areas is involved in inhibiting distractors. This interpretation is in accordance with some results reported in attention studies (see Working Memory) highlighting the close relationship between WM and attention.

The studies presented above have all focused on the maintenance phase of the WM. Kaplan et al. (2014) focused on the retrieval phase of spatial memory. The phase coupling was assessed by calculating PLV between a seed voxel in the medial Pre-frontal Cortex (mPFC) and all the other voxels in the brain. Significant increase (compared to baseline) in theta coupling was found between right anterior medial temporal lobe (aMTL) and the seed voxel and this theta coupling was argued to play a role in mnemonic functions. The phase coupling was also analyzed with PLI and this analysis confirmed that the increase in theta coupling was not an artifact of volume conduction. The PLI analysis also showed increased theta coupling between the seed and nearby anterior cingulate cortex. However, the authors acknowledged that the phase coupling between so nearby regions should be interpreted with caution (Kaplan et al., 2014). The evidence indicates that phase coupling within different frequency bands plays a role in WM processes. Siebenhühner et al. (2016) expanded the perspective by showing that during the maintenance period of DMS task, cross-frequency coupling (assessed by PLV^*n*:*m*^) was enhanced above baseline between high-alpha and beta and gamma band oscillations. Similarly, cross-frequency coupling between high-theta and alpha, beta and gamma band oscillations were enhanced in contrast to baseline. The strength of the cross-frequency coupling of theta and high-alpha with their corresponding higher frequencies was also positively correlated with the VWM load and predicted inter-individual variability in VWM capacity. The hubs characterized by low-frequency oscillation were located predominantly in the frontoparietal and to a lesser extent in dorsal attention areas. The low frequency oscillations exhibited by these hubs were coupled with faster oscillations in the visual system and dorsal attention areas. In contrast, the higher frequency oscillations had hubs pre-dominantly in dorsal attention areas and in the visual cortex (gamma band). Moreover, the most central hubs involved in the cross-frequency coupling were co-localized with the most central hubs involved in the slow and fast within frequency coupling. These results suggest that cross-frequency coupling connects the visual system and attentional networks and therefore integrates the representational and central executive functions of VWM (Siebenhühner et al., 2016).

Taken together, various MEG studies have shown that phase coupling within different frequency bands plays a role in WM memory processes. Phase coupling in alpha band is the most frequently reported observation and it is linked to inhibiting distractors (Wianda and Ross, 2019) and attentional functions (Palva et al., 2010; Sato et al., 2018). Phase coupling in beta and gamma bands on the other hand is linked to binding neuronal representations, a process in which IPS could be the key hub between the visual and frontoparietal networks (Palva et al., 2010). Furthermore, cross-frequency coupling could be the mechanism which integrates different functional systems together (Siebenhühner et al., 2016).

Interestingly, all the WM studies referred made contrast only between one working memory state and baseline/control. This approach can lead to an illusion that observed phase coupling networks are dedicated solely to the WM state that has been studied. This combined with a-priori selection of seed regions or frequency bands can lead to a conclusion where single or few functional interactions are dedicated to the function of interest. An fMRI study (Soreq et al., 2019) recently showed, by contrasting a broad range of behavioral conditions, that different aspects of WM were characterized by heavily overlapping multivariate activation and connectivity patterns.

## Conclusions

In the last decade, the increasing body of work dealing with MEG functional connectivity analysis and with method development has demonstrated the efficacy of this technique in assessing the coupling between brain regions and in characterizing brain networks.

In this review, we focused on data-driven methods to estimate functional connectivity based on phase coupling by MEG. All of these methods are here formulated for static connectivity analysis, which requires averages over a theoretically infinite number of data segments. In practice, data length is always finite, and the reliability of these methods is impacted by the overall number of available data time points. This issue has been discussed in simulated data for linear coupling methods (e.g., Sommariva et al., 2017), while for cross-frequency coupling methods this is still to be explicitly investigated. Moreover, to date several evidences have been provided concerning the changes of functional connectivity patterns across different time-scales (Breakspears et al., 2004). The extension of the above described methods to the study of dynamical phase coupling is straightforwardly achievable through the use of the Time-Frequency resolved versions of these metrics, at least for task-induced connectivity.

Besides the above reviewed methods, there is a wide family of connectivity methods which could be used to assess functional or effective couplings between neuronal oscillations, both at the same or at different frequencies. These include, but are not limited to: phase-amplitude coupling methods, which assess the coupling between the phase of a low-frequency oscillation and the amplitude of a high-frequency oscillation (Canolty et al., 2006; Cohen, 2008; Tort et al., 2010; Özkurt and Schnitzler, 2011); amplitude-amplitude coupling methods, which investigate the covariation of aperiodic fluctuations such as the amplitudes of brain oscillations at a specific frequency (Brookes et al., 2011b; Hipp et al., 2012); model-based approaches, such as the dynamic causal models (Friston et al., 2003), which infer the causal structure of brain connections by relying on Bayesian inference; methods to assess the directionality of interactions based on the concept of Granger causality (Geweke, 1982; Kaminski and Blinowska, 1991; Sameshima and Baccalá, 1999; Marinazzo et al., 2008). In addition, methods to identify brain states can provide further insight into brain networks and especially into their dynamics. In this framework, microstate analysis identifies sensor level topographies that remain stable for a certain period of time before transitioning to a different topography. Changes in the topography are assumed to indicate a reorganization in the global coordination of neuronal activity over time. This approach has a long lasting tradition in EEG, but it has seen a new flourishing in the recent years due to close link to brain network analysis. For timely reviews see Michel and König (2018) and He et al. (201. Similarly, Hidden Markov Model (HMM) approaches reveal the transition of brain networks between states that recur at different points in time. This approach has been used in MEG source space data in Baker et al. (2014) for network identification through amplitude based correlation, and in Vidaurre et al. (2018) to identify phase coherence based networks. A more detailed description of all these methods for assessing brain connectivity and brain networks would require a dedicated review paper and it is thus beyond the scope of this work.

There are a number of methodological issues and open questions concerning connectivity estimation which need to be addressed. For instance, the question of whether increases in connectivity might be merely driven by increases in the amplitude of oscillatory activity, or whether the connectivity itself might induce increases in local amplitudes is still debated (Daffertshofer and van Wijk, 2011; Moon et al., 2015; Tewarie et al., 2019). Another methodological issue is that all connectivity methods, including those which have been designed to specifically handle artificial connectivity detections due to zero-lag correlations, are not safe from spurious connections caused by source leakage in the proximity of true interactions, i.e., the so-called “ghost interactions” (Palva et al., 2018). Finally, the relationship between some coupling measures and the underlying physiological mechanisms is not well-understood. For instance, cross-frequency coupling measures might be influenced by features in the signal that do not relate to interactions, such as the non-sinusoidal waveform of brain oscillations which generate the coupling between harmonically related frequencies (Aru et al., 2015; Lozano-Soldevilla et al., 2016; Deco et al., 2017).

In this review, we also show examples of applications of phase coupling methods to MEG data from different cognitive domains. Of note, in these studies, connectivity results were disentangled from power results proving evidence for a genuine phase coupling between brain areas. Moreover, in the resting state and in the visuospatial attention examples, the MEG connectivity results have been supported by MRI functional or anatomical connectivity. While the benefits of integrating different modalities in the study of functional connectivity by MEG is self-evident, still actual multimodal connectivity analysis tools are lacking. Along the same line, a comprehensive characterization of the benefits and/or limitations in the integration procedures is missing. One example of integrating MEG functional connectivity with MRI information is the recent characterization of the impact of MEG-MRI coregistration errors in functional connectivity estimated through different metrics (Chella et al., 2019).

In conclusion, we believe that the field has made substantial steps forward in the recent years and is now ready for bringing the study of functional connectivity toward a more mechanistic understanding of the role of brain rhythms, also aided by high-level modality integration tools for connectivity.

## Author Contributions

LM and VP contributed to the conception and design of the review and wrote the first draft of the manuscript. AB, FC, AD'A, and JS wrote the sections of the manuscript. All authors contributed to manuscript revision, read, and approved the submitted version.

### Conflict of Interest Statement

The authors declare that the research was conducted in the absence of any commercial or financial relationships that could be construed as a potential conflict of interest.
